# Supplementary material for: Analysis of the 24-h activity cycle: An illustration examining the association with cognitive function in the Adult Changes in Thought study
Source: Front Psychol. 2023 Mar 27;14:1083344. doi: 10.3389/fpsyg.2023.1083344 (PMC10087899; doi:10.3389/fpsyg.2023.1083344)
Supplement: Supplementary file 1 [file Data_Sheet_1.docx]

**Supplemental Web Materials for**

**Analysis of the 24-Hour Activity Cycle: An illustration examining the association with cognitive function-in the Adult Changes in Thought (ACT) Study**

Yinxiang Wu^1,2^, Dori E. Rosenberg^1^, Mikael Anne Greenwood-Hickman,^1^ Susan M. McCurry^2^, Cecile Proust-Lima^3^, Jennifer C. Nelson^1^, Paul K. Crane^2^, Andrea LaCroix,^4^ Eric B. Larson,^2^ Pamela A. Shaw^1^*

1. Kaiser Permanente Washington Research Institute, Seattle, WA, USA
2. University of Washington, Seattle, WA, USA
3. Bordeaux Population Health Research Center, INSERM, Bordeaux, France
4. Herbert Wertheim School of Public Health and Human Longevity Science, University of California, San Diego, CA, USA

**A1. Compositional Data Analysis (CoDA) Supplemental Methods and Results**

**A1.1 Definition of “addition” and “multiplication” operations in the simplex space and their connection to the definition of compositional mean**

The collection of all D-part compositions $S^{D}= \{\left[ x_{1},..,x_{D} \right]:x_{i}>0 \left( i=1,\ldots,D \right), x_{1}+\ldots+x_{D}=1\}$ is called the simplex space. Aitchison (1986) defined two fundamental operations, namely perturbation and power operations, in the simplex space $S^{D}$ analogous to the addition and multiplication operations in real space. The perturbation operator denoted as ⊕ can be used to characterize 'difference' between compositions or change from one composition to another. In formula, the perturbation operation is defined as follows.

Consider two compositions $\mathbf{x} = [x_{1}, \ldots, x_{D}]$ and $\boldsymbol{y =}[y_{1}, \ldots, y_{D}]$,

$$\boldsymbol{x}\oplus\boldsymbol{y} = \boldsymbol{C}[x_{1}y_{1}, \ldots, x_{D}y_{D}]\boldsymbol{=}[x_{1}y_{1}, \ldots, x_{D}y_{D}]/(x_{1}y_{1} + \ldots+ x_{D}y_{D})$$

where **C** is the so-called *closure* operation which divides each component of a composition by the sum of its components so as to scale the composition to the constant sum 1. It is not hard to see that the identity perturbation induced by this operation is $[\frac{1}{D}, \ldots,\frac{1}{D}]$ (meaning that every composition perturbed by this identity will remain the same, playing the same role as 0 in the real space) and the inverse of a perturbation denoted as Θ is given by $\boldsymbol{x} \Theta\boldsymbol{y = C}[\frac{x_{1}}{y_{1}},\ldots,\frac{x_{D}}{y_{D}}].$As as consequence, the change from **x** to **y** is expressed by the perturbation **y** Θ **x**.

The operation analogous to scalar multiplication in real space is the power operation denoted as ⊗. For any real number a in R, and any composition **x** in $S^{D}$, the $a$-power transform of **x** is defined to be

$$a\otimes\boldsymbol{x = C}[x_{1}^{a},\ldots.,x_{D}^{a}]$$

By drawing analogy with the definition of sample mean in real space i.e. mean = $\frac{1}{n} \sum(x_{1}+\ldots+x_{n})$, we can define the compositional mean in a similar way but by using the operations defined on the simplex space $S^{D}$. Suppose we observe n compositions in $S^{D}$, labelled as $\boldsymbol{x}_{1},\ldots,\boldsymbol{x}_{n}$. By applying the same sample mean formula with replacement of every addition and multiplication operation in real space by the perturbation and power operation, we get $(\frac{1}{n}) \otimes(\boldsymbol{x}_{\boldsymbol{1}}\oplus\boldsymbol{x}_{\boldsymbol{2}}\oplus\ldots\oplus\boldsymbol{x}_{n})$ which is exactly the definition of compositional mean provided in the main text. Furthermore, if the “Aitchison distance” (Aitchison et al., 2000) is used as a metric of distance on the simplex space, it can be further shown that the compositional mean minimizes the “Aitchison distance” between all data points.

**A1.2 Sequential binary partition procedure to create ilr-coordinates and examples**

The sequential binary partition process (SBP) (Egozcue et al., 2005) makes it easy to create *ilr*-coordinates. In the first step of the process, all parts are split into two groups and a coordinate is defined to be the log-ratio between the geometric mean of the two groups. In the following steps, each group in the previous ratio, is in turn split into two groups and the log-ratio between the geometric mean of the two groups is another coordinate, and the process continues until the two groups in the ratio each have a single component. A typical *ilr*-transformation for 24HAC keeps a single behavior in the numerator at each step, as shown in the example (equation 2) in main text. This process can be visualized as the table below.

| Level of partition | Sit | Stand | Step | Sleep | r | s |
| --- | --- | --- | --- | --- | --- | --- |
| 1 | + | - | - | - | 1 | 3 |
| 2 |  | + | - | - | 1 | 2 |
| 3 |  |  | + | - | 1 | 1 |

r: the size of numerator group (labelled as “+”); s: the size of denominator group (labelled as “-”)

The formula to calculate the *ilr*-coordinate resulted from each level of partition is $z= \sqrt{\frac{rs}{r+s}}ln(\frac{N}{D})$ where $N, D$ represent, respectively, the geometric mean of the activities involved in the numerator and denominator group.

Another other example of *ilr*-coordinates is as follows:

$$z_{1}= ln\frac{{(Stand\times Step)}^{1/2}}{{(Sit\times Sleep)}^{1/2}}$$

$$z_{2}= \sqrt{\frac{1}{2}}ln\frac{Stand}{Step}$$

$$z_{3}= \sqrt{\frac{1}{2}}ln\frac{Sit}{Sleep}$$

The table for the sequential binary process of generating this set of *ilr*-coordinates is

| Level of partition | Sit | Stand | Step | Sleep | r* | s* |
| --- | --- | --- | --- | --- | --- | --- |
| 1 | - | + | + | - | 2 | 2 |
| 2 |  | + | - |  | 1 | 1 |
| 3 | + |  |  | - | 1 | 1 |

* r: the size of numerator group (labelled as “+”); s: the size of denominator group (labelled as “1”)

The specific set of *ilr*-coordinates can be chosen to capture a particular comparison of geometric means to aid interpretation of that coordinate. For example, if the goal is to estimate the effect of increasing time in both stand and step while simultaneously decreasing time in sit and sleep on an outcome, then by regressing the outcome on the $z_{1}$, $z_{2}$ and $z_{3}$ defined as above, this effect is captured by the regression coefficient for $z_{1}$ (as $z_{2}$ and $z_{3}$ are held constant).

**A1.3 Computation of the associated change in the mean outcome between any two given compositions**

Consider two example compositions that were used for illustration purpose in the main text: namely, 10h (41.7%) sitting, 3h (12.5%) standing, 2h (8.3%) stepping, and 9h (37.5%) sleeping per day, and 7.6h (31.7%) sitting, 5.4h (22.5%) standing, 2h (8.3%), and 9h (37.5%) sleeping per day, denoted as $\boldsymbol{x}_{\boldsymbol{1}}$ and $\boldsymbol{x}_{\boldsymbol{2}}$, respectively. By using the perturbation and power operations defined in A1.1, the change from $\boldsymbol{x}_{\boldsymbol{1}}$ to $\boldsymbol{x}_{\boldsymbol{2}}$ equals $\boldsymbol{x}_{2} \Theta\boldsymbol{x}_{1}$. The *ilr*-transformation of $\boldsymbol{x}_{2} \Theta\boldsymbol{x}_{1}$ is *ilr*($\boldsymbol{x}_{2}$) – *ilr*($\boldsymbol{x}_{1}$) because ilr-transformation is an isometric isomorphism between $S^{4}$ and $R^{3}$ (Pawlowsky-Glahn et al., 2015). If we denote *ilr*($\boldsymbol{x}_{1}$) as $P_{1}$ and *ilr*($\boldsymbol{x}_{2}$) as $P_{2}$ and the *ilr*-coordinates defined in the equation (3) in the main text are used, then $P_{1}$ and $P_{2}$ are just two points in $R^{3}$ with $P_{1}$ = (-1.02, 0.53, -0.78) and $P_{2}$ = (-1.12, 0.06, -0.34), and the change from $\boldsymbol{x}_{\boldsymbol{1}}$ to $\boldsymbol{x}_{\boldsymbol{2}}$ can be characterized by the change from $P_{1}$ to $P_{2}$ with difference of -0.1, -0.48, and 0.44 in each coordinate $z_{1}$, $z_{2}$, and $z_{3}$. With a fitted linear model $\hat{E}(Y)= \hat{\beta}_{0}+ \hat{\beta}_{1} z_{1}+ \hat{\beta}z_{2}+ \hat{\beta}_{3}z_{3}$, the effect of time reallocation corresponding to the change from $\boldsymbol{x}_{\boldsymbol{1}}$ to $\boldsymbol{x}_{\boldsymbol{2}}$ is then -0.1$\hat{\beta}_{1}$-0.48$\hat{\beta}_{2}$+0.44$\hat{\beta}_{3}$.

**A2. Supplemental Methods and Materials for Latent Profile Analysis**

**A2.1. Underlying probabilistic model in LPA**

Suppose we observe three continuous variables ($X_{1}, X_{2}, X_{3}$) and assume there are $K$ hidden profiles (call each profile $G_{k}$, $k=1,\ldots,K$) underlying the observed data. LPA assumes the likelihood of any observation from the data follows

$$P\left( x_{1}, x_{2}, x_{3} \right)=\sum_{k=1}^{K} P\left( G_{k} \right)P\left( x_{1},x_{2},x_{3}|G_{k} \right)$$

where $P\left( G_{k} \right)$ is the probability of observing latent profile $G_{k}$, k = 1, …. K; $P\left( x_{1},x_{2},x_{3}|G_{k} \right)$ is the probability density function for a multivariate normal distribution $N(\mu_{k}, \Sigma_{k})$, k=1,…,K with ith profile specific mean vector $\mu_{i}$ and covariance matrix $\Sigma_{k}$. In this framework, $P\left( G_{k} \right)$, $\mu_{k}$, $\Sigma_{k}$ are all unknown quantities for each profile. If they are known or can be estimated from the data, we can then compute $P\left( G_{k}|x_{1},x_{2},x_{3} \right)$ the probability of an observation belonging to a latent profile $G_{k}$ (or called posterior probability) by using Bayes rule

$P\left( G_{k}|x_{1}, x_{2}, x_{3} \right)=\frac{P\left( G_{k} \right)P\left( x_{1},x_{2},x_{3}|G_{k} \right)}{\sum_{k=1}^{K} P\left( G_{k} \right)P\left( x_{1}, x_{2},x_{3}|G_{k} \right)}$ for each profile k = 1,…,K.

The key components of LPA are to determine (1) the number of underlying latent profiles, and (2) variance-covariance structures (e.g. whether variances and covariances could vary between profiles) for the multivariate normal distributions, both of which can be tuned by using the fit statistics and other criteria we discussed in the main text.

Although it is generally assumed that mean vectors differ across profiles, LPA model can also vary in terms of how the class-specific (co)variance matrices of the indicator variables are constrained or allowed to vary within and between classes. The four commonly used models are listed below (Wardenaar 2021):

1. The variances of individual profile indicators are set to be the same across classes but the covariances between them are set to be 0 (i.e. independent under the normal assumption)
2. Both the variances and covariances of profile indicators are set to be the same across classes, but the covariances can be non-zero.
3. The variances of profile indicators can differ between classes but the covariances are set to be 0.
4. Both the variances and covariances can differ between classes and the covariances can be non-zero (most flexible model).

**A2.2. Latent Gold 6.0 Syntax for step 3 adjusted association analysis of latent profiles with CASI IRT score using improved BCH method to account for class uncertainty**

**
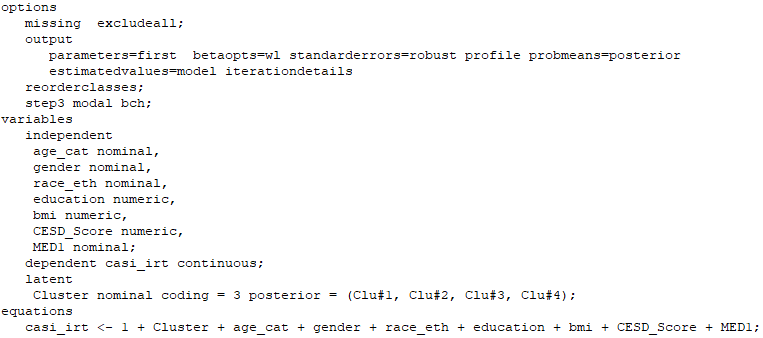
**

**References**

Aitchison, J. The Statistical Analysis of Compositional Data. Chapman & Hall: London, UK, 1986; Reprinted in 2003 by Blackburn Press; p. 416.

Aitchison J, Barceló-Vidal C, Martín-Fernández JA, Pawlowsky-Glahn V. Logratio analysis and compositional distance. Mathematical Geology. 2000 Apr;32(3):271-5.

Egozcue JJ, Pawlowsky-Glahn V. Groups of parts and their balances in compositional data analysis. Mathematical Geology. 2005 Oct;37(7):795-828.

Pastor DA, Barron KE, Miller BJ, Davis SL. A latent profile analysis of college students’ achievement goal orientation. Contemporary Educational Psychology. 2007 Jan 1;32(1):8-47.

Pawlowsky-Glahn V, Egozcue JJ, Tolosana-Delgado R. Modeling and analysis of compositional data. John Wiley & Sons; 2015 Mar 30.

Wardenaar K. Latent Profile Analysis in R: A tutorial and comparison to Mplus. 2021 Apr 9. PrePrint doi 10.31234/osf.io/wzftr.

**A.3 Supplemental Tables**

**Table S1. Variation matrix for the 24HAC composition of sit, stand, step and sleep (N=1034).^1^**

|  | Sit | Stand | Step | Sleep |
| --- | --- | --- | --- | --- |
| Sit | 0 | 0.43 | 0.54 | 0.08 |
| Stand | 0.43 | 0 | 0.23 | 0.27 |
| Step | 0.54 | 0.23 | 0 | 0.42 |
| Sleep | 0.08 | 0.27 | 0.42 | 0 |

^1^Each entry is the standard deviation of the logarithm of the ratio between two activities in the sample.

**Table S2 Comparisons of the 24-hour Activity compositional mean by participant characteristics (N = 1034).** P-values correspond to the James multivariate analysis of variance test comparing the means of *ilr*-transformed data, assuming unequal variance between groups. The number of observations in subgroups may not sum to 1034 due to missing values.

|  | N (%) | | Sitting,  h/day (%) | Standing,  h/day (%) | Stepping,  h/day (%) | Sleeping,  h/day (%) | p-value |
| --- | --- | --- | --- | --- | --- | --- | --- |
| Overall | 1034 (100%) | 10.2 (42.6%) | | 3.7 (15.3%) | 1.2 (5.2%) | 8.8 (36.9%) |  |
| Gender |  |  | |  |  |  | <0.001 |
| Male | 457 (44.2%) | 10.6 (44.2%) | | 3.4 (14.2%) | 1.3 (5.3%) | 8.7 (36.3%) |  |
| Female | 577 (55.8%) | 9.9 (41.2%) | | 4.0 (16.5%) | 1.3 (5.2%) | 8.9 (37.1%) |  |
| Age |  |  | |  |  |  | <0.001 |
| 65-74 | 433 (41.9%) | 10.0 (41.5%) | | 3.9 (16.2%) | 1.5 (6.3%) | 8.6 (36%) |  |
| 75-84 | 425 (41.1%) | 10.2 (42.4%) | | 3.7 (15.5%) | 1.2 (5.2%) | 8.9 (37%) |  |
| 85+ | 176 (17%) | 10.8 (45.1%) | | 3.2 (13.5%) | 0.9 (3.6%) | 9.1 (37.8%) |  |
| Race/Ethnicity^1^ |  |  | |  |  |  | 0.002 |
| Non-Hispanic White | 920 (89%) | 10.2 (42.5%) | | 3.7 (15.3%) | 1.3 (5.3%) | 8.9 (36.9%) |  |
| Hispanic/Other race | 109 (0.5%) | 10.3 (42.8%) | | 3.9 (16.4%) | 1.1 (4.7%) | 8.6 (36.1%) |  |
| Self-rated Health |  |  | |  |  |  | <0.001 |
| Excellent/Very good/Good | 952 (92.1%) | 10.1 (42.2%) | | 3.8 (15.7%) | 1.3 (5.5%) | 8.8 (36.5%) |  |
| Fair/Poor | 82 (7.9%) | 11 (45.9%) | | 2.8 (11.8%) | 0.7 (3.1%) | 9.4 (39.2%) |  |
| Physical function score^1^ |  |  | |  |  |  | <0.001 |
| <= 10 | 723 (69.9%) | 10.3 (42.9%) | | 3.7 (15.3%) | 1.2 (5.1%) | 8.8 (36.7%) |  |
| >10 | 243 (23.5%) | 9.7 (40.6%) | | 4.0 (16.5%) | 1.6 (6.7%) | 8.7 (36.3%) |  |
| Ability to walk half mile^1^ |  |  | |  |  |  | <0.001 |
| No difficulty | 771 (74.6%) | 9.9 (41.1%) | | 4 (16.5%) | 1.5 (6.2%) | 8.7 (36.2%) |  |
| Some difficulty | 149 (14.4%) | 10.7 (44.4%) | | 3.4 (14.1%) | 0.9 (3.9%) | 9 (37.6%) |  |
| A lot difficulty/Unable | 110 (10.6%) | 11.6 (48.5%) | | 2.5 (10.5%) | 0.6 (2.6%) | 9.2 (38.4%) |  |
| Sleep duration/day |  |  | |  |  |  | <0.001 |
| <6 hrs | 17 (1.6%) | 12.3 (51.3%) | | 4.5 (18.6%) | 1.3 (5.3%) | 5.9 (24.7%) |  |
| 6-9 hrs | 669 (64.7%) | 10.4 (43.5%) | | 3.9 (16.2%) | 1.4 (5.7%) | 8.3 (34.7%) |  |
| 9+ hrs | 348 (33.7%) | 9.6 (40%) | | 3.3 (13.8%) | 1.1 (4.5%) | 10 (41.7%) |  |
| Sleep quality^1^ |  |  | |  |  |  | 0.034 |
| Very poor to fair | 342 ( 33.1%) | 10.4 (43.2%) | | 3.6 (15%) | 1.2 (5%) | 8.8 (36.8%) |  |
| good to very good | 596 (57.6%) | 10.1 (41.9%) | | 3.8 (15.9%) | 1.3 (5.6%) | 8.8 (36.6%) |  |
| CASI IRT score |  |  | |  |  |  | <0.001 |
| <= 0 | 198 (19.1%) | 10.6 (44%) | | 3.4 (14.3%) | 1.1 (4.4%) | 9 (37.3%) |  |
| (0, 1] | 506 (48.9%) | 10.2 (42.4%) | | 3.7 (15.5%) | 1.3 (5.3%) | 8.8 (36.8%) |  |
| >1 | 330 (31.9%) | 10 (41.8%) | | 3.8 (16%) | 1.4 (5.8%) | 8.7 (36.4%) |  |

^1^ Missing data: race/ethnicity N = 5; physical function N = 68; ability to walk half a mile N = 4; sleep quality N = 96.

**Table S3. Fit statistics for latent profile models with 2-6 profiles. Bold font numbers indicate the best results in terms of each fit statistic (N = 1034).^1^**

| Class  Number | LogLik | AIC | BIC | CAIC | SABIC | ICL-BIC | N  min (%) | LMR  p-value | BLRT  p-value | Entropy^2^ |
| --- | --- | --- | --- | --- | --- | --- | --- | --- | --- | --- |
| 2 | 5595.1 | -11152.3 | -11058.4 | -11039.4 | -11118.8 | **-10337.8** | 365 (35.3%) |  |  | 0.50 |
| 3 | 5648.8 | -11239.6 | **-11096.4** | **-11067.4** | -11188.5 | -10118.8 | 177 (17.1%) | <0.001 | <0.001 | 0.57 |
| 4 | 5673.5 | -11269.1 | -11076.4 | -11037.4 | **-11200.2** | -9784.0 | 132 (12.8%) | <0.001 | <0.001 | 0.55 |
| 5 | 5688.4 | -11278.8 | -11036.6 | -10987.6 | -11192.3 | -9550.4 | 80 (7.7%) | 0.022 | <0.001 | 0.55 |
| 6 | 5701.7 | **-11285.4** | -10993.9 | -10934.9 | -11181.3 | -9581.9 | 46 (4.4%) | 0.103 | <0.001 | 0.62 |

^1^ Abbreviations: AIC (Akaike’s Information Criterion), BIC (Bayesian Information Criteria), CAIC (Consistent Akaike’s Information Criterion), SABIC (sample adjusted Bayesian Information Criteria), ICL-BIC (integrated complete likelihood-Bayesian Information Criteria), LMR (Lo-Mendell-Rubin likelihood ratio test), BLRT (Bootstrap likelihood ratio test).

^2^ Entropy statistic ranges from 0 to 1 with higher values indicative of lower uncertainty in profile classification; 1 means perfect classification (i.e., without uncertainty) (Pastor et al., 2007). For example, when there are K latent classes, the formula to calculate this entropy based on N observations with each observation having probabilities $p_{1}$, …,$p_{K}$ belonging to class 1 to class K, respectively, is

$1- \frac{\sum_{i=1}^{N} \sum_{k=1}^{K} (-p_{ik}log(p_{ik}))}{Nlog(K)}$.

**Table S4. Estimated profile-specific mean and variance-covariance matrix of the three profile indicators (sit, stand, step) in the final 4-class model (N = 1034).**

|  | Profile 1, most active (15.9%^1^) | Profile 2, moderately active low sleeper (24.4%^1^) | Profile 3, average activity (40.3%^1^) | Profile 4, least active (19.4%^1^) |
| --- | --- | --- | --- | --- |
| **Mean (hrs/day)** |  |  |  |  |
| Sit | 7.6 | 9.6 | 10.3 | 12.1 |
| Stand | 5.8 | 4.7 | 3.5 | 2.4 |
| Step | 1.9 | 1.7 | 1.3 | 0.7 |
| Sleep^2^ | 8.7 | 7.9 | 8.9 | 8.8 |
| **SD (hrs/day)** |  |  |  |  |
| Sit | 1.4 | 1.6 | 1.3 | 1.7 |
| Stand | 1.8 | 1.1 | 0.9 | 1.0 |
| Step | 0.8 | 0.5 | 0.4 | 0.3 |
| Sleep^2^ | 1.1 | 1.0 | 0.6 | 1.4 |
| **Correlation** |  |  |  |  |
| Sit-Stand | -0.8 | -0.7 | -0.8 | -0.6 |
| Sit-Step | -0.1 | -0.3 | -0.5 | -0.4 |
| Stand-Step | -0.2 | -0.2 | 0.3 | 0.7 |
| Sit-Sleep^2^ | <0.01 | -0.15 | -0.09 | -0.17 |
| Stand-Sleep^2^ | -0.11 | 0.01 | 0.02 | -0.05 |
| Step-Sleep^2^ | -0.07 | 0.05 | <0.01 | -0.06 |

^1^ Estimated probability of each profile

^2^ Sleep was not included in the LPA analyses. However, because the proportions of four activities sum to 1, the SD of sleep and its correlation with other activities can be derived. For example, the variance of sleep equals var(1-sit-stand-step) = var(sit + stand + step) = var(sit) + var(stand) + var(step) + 2cov(sit, stand) + 2 cov(sit, step) + 2 cov(stand, step).

**Table S5. Expected misclassification errors for modal assignment based on the final 4-class LPA model presented in the main text (N = 1034).^1^**

|  | **Profile according to modal assignment** | | | |
| --- | --- | --- | --- | --- |
| **Latent profile** | **1** | **2** | **3** | **4** |
| **1** | 0.797 | 0.103 | 0.089 | 0.012 |
| **2** | 0.215 | 0.669 | 0.017 | 0.010 |
| **3** | 0.216 | 0.036 | 0.747 | 0.001 |
| **4** | 0.142 | 0.233 | 0.007 | 0.618 |

^1^ Each entry is the estimated probability that an observation belonging to a latent profile (indicated by row number) is assigned to each profile (indicated by column number).

**A.4 Supplemental Figures**

**Figure S1. Scatterplots of data points in the simplex space for the 3-part composition of sit, stand, and step (left graph), and in 2D real space with isometric logratio coordinates** $\boldsymbol{z}_{\boldsymbol{1}}$ **and** $\boldsymbol{z}_{\boldsymbol{2}}$**.** $\boldsymbol{z}_{\boldsymbol{1}}\boldsymbol{=}\sqrt{\frac{\boldsymbol{2}}{\boldsymbol{3}}}\mathbf{ln}\frac{\boldsymbol{Sit}}{\boldsymbol{(Stand\times Step)}}$ **and** $\boldsymbol{z}_{\boldsymbol{2}}\boldsymbol{=}\sqrt{\frac{\boldsymbol{1}}{\boldsymbol{2}}}\mathbf{ln}\frac{\boldsymbol{Stand}}{\boldsymbol{Step}}$ **(right graph).** The red dot on each graph below is an example data point located in the simplex space (before *ilr*-transformation) and 2D real space (after *ilr*-transformation), respectively.


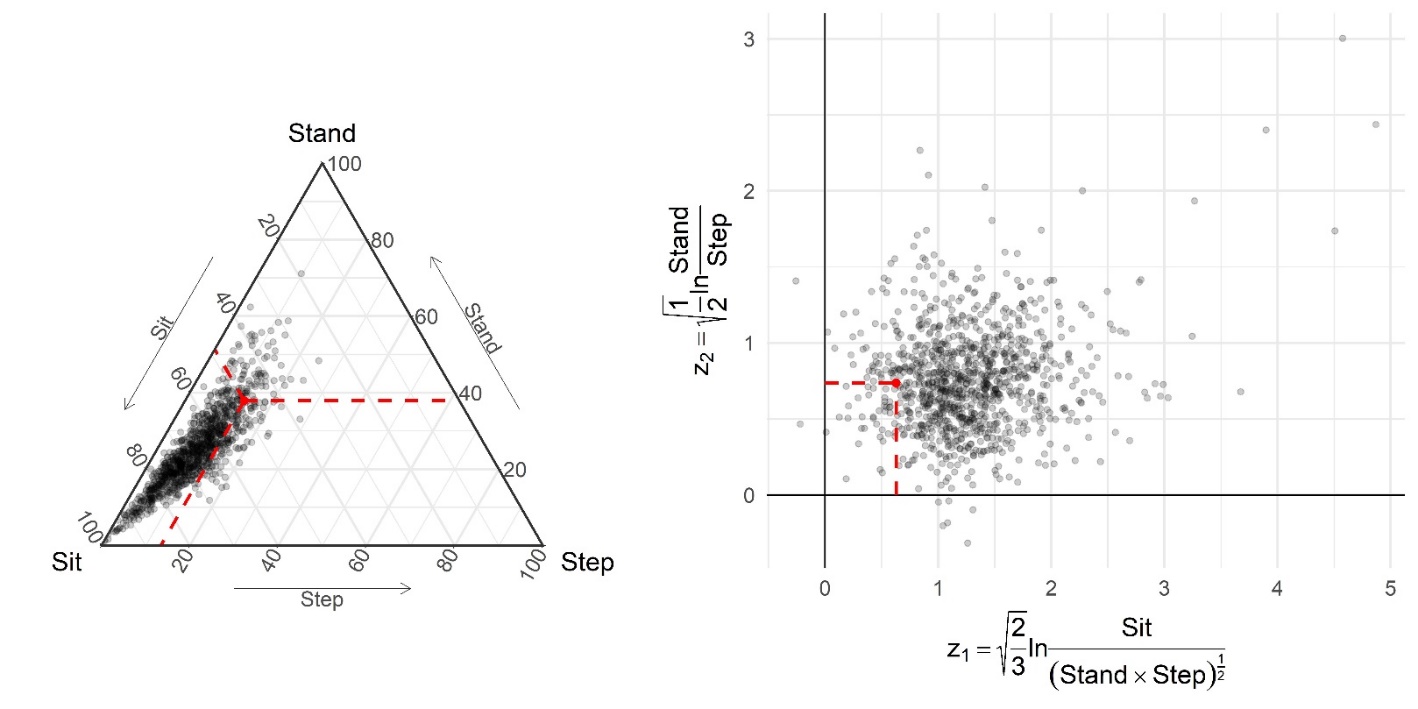


**Figure S2. Distributions of percentage time per day spent in each behavior (N=1034).** Upper triangular entries are Pearson’s correlation coefficients. Superscript stars indicate the level of significance i.e. *** indicates p-value < 0.001.


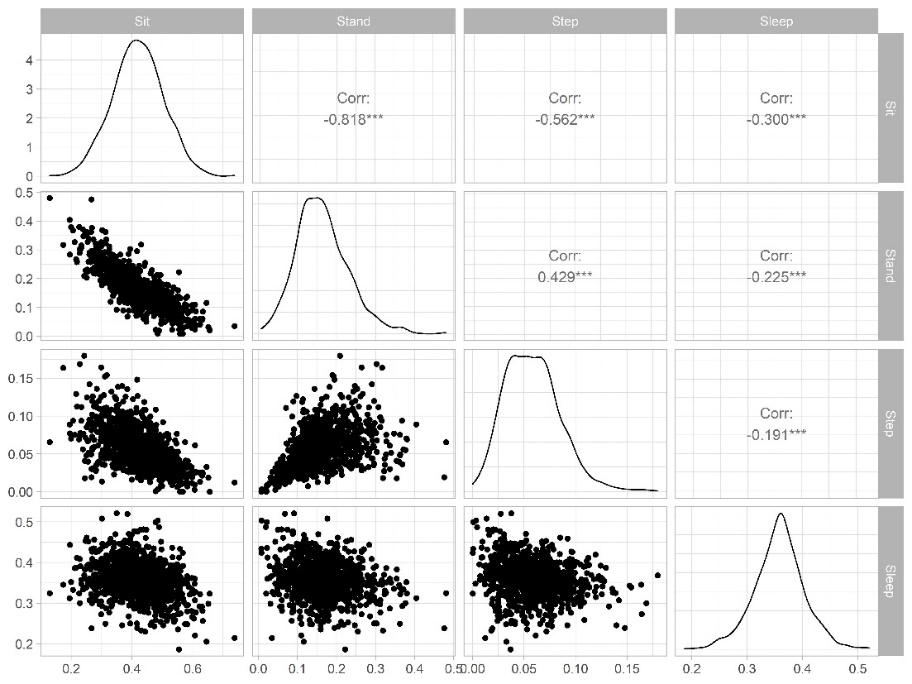


**Figure S3. Distributions of *ilr*-transformed data with ilr-coordinates** $\boldsymbol{z}_{\boldsymbol{1}}$**,** $\boldsymbol{z}_{\boldsymbol{2}}$**,** $\boldsymbol{z}_{\boldsymbol{3}}$ **specified in equation (2) in the main manuscript, Section 3.2 (N=1034).** Upper triangular entries are Pearson’s correlation coefficients. Superscript stars indicate the level of significance i.e. *** indicates p-value < 0.001; ** indicates p-value <0.01.


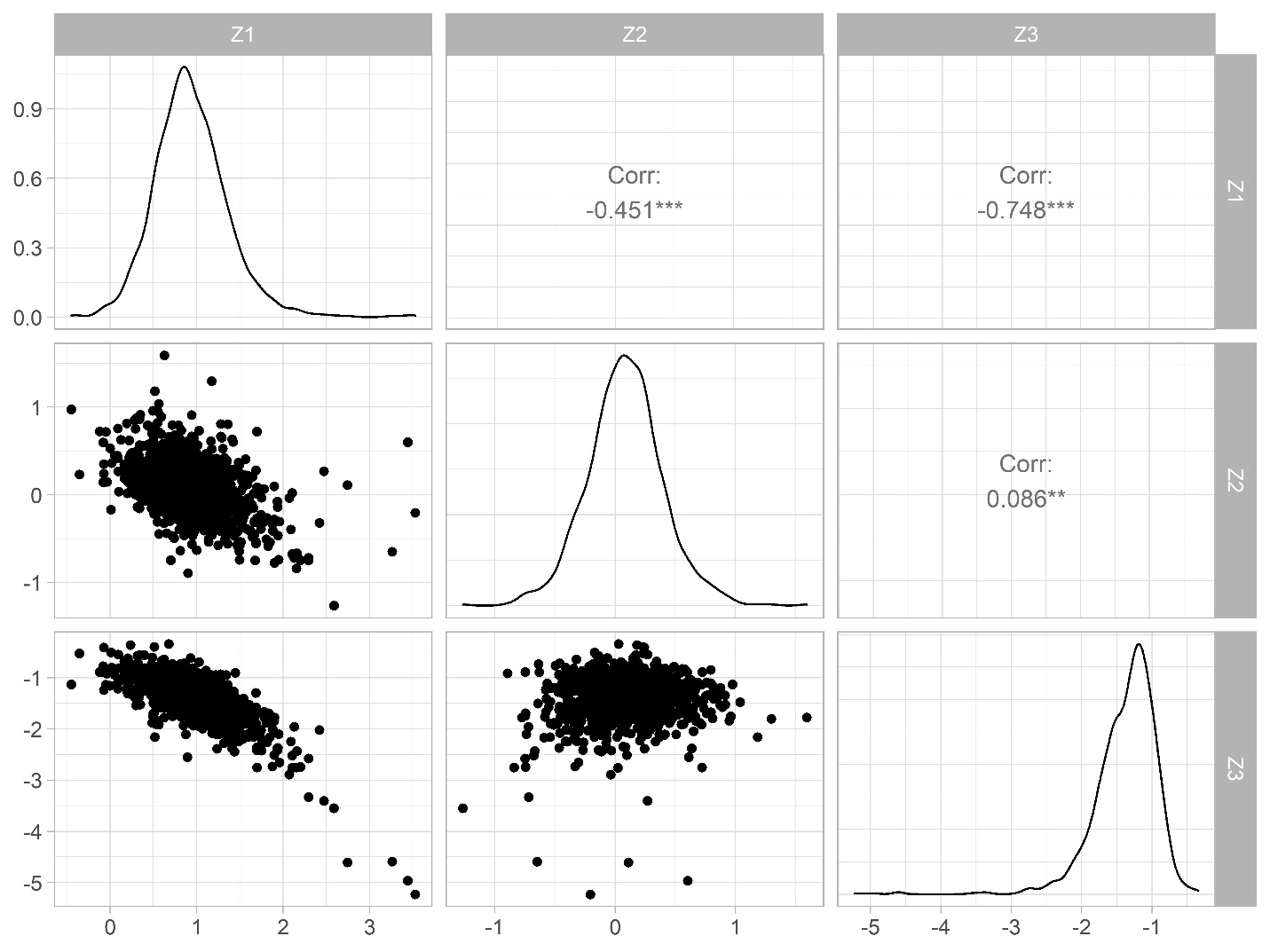


**Figure S4. Fitted 24HAC profiles from latent profile analysis (3-class solution), where each individual is assigned the class with maximum probability. The boxplots present sample quartiles (N=1034).** The number (%) of subjects in profile 1-3 is, respectively, 177 (17.1%), 622 (60.2%), and 235 (22.7%).

**
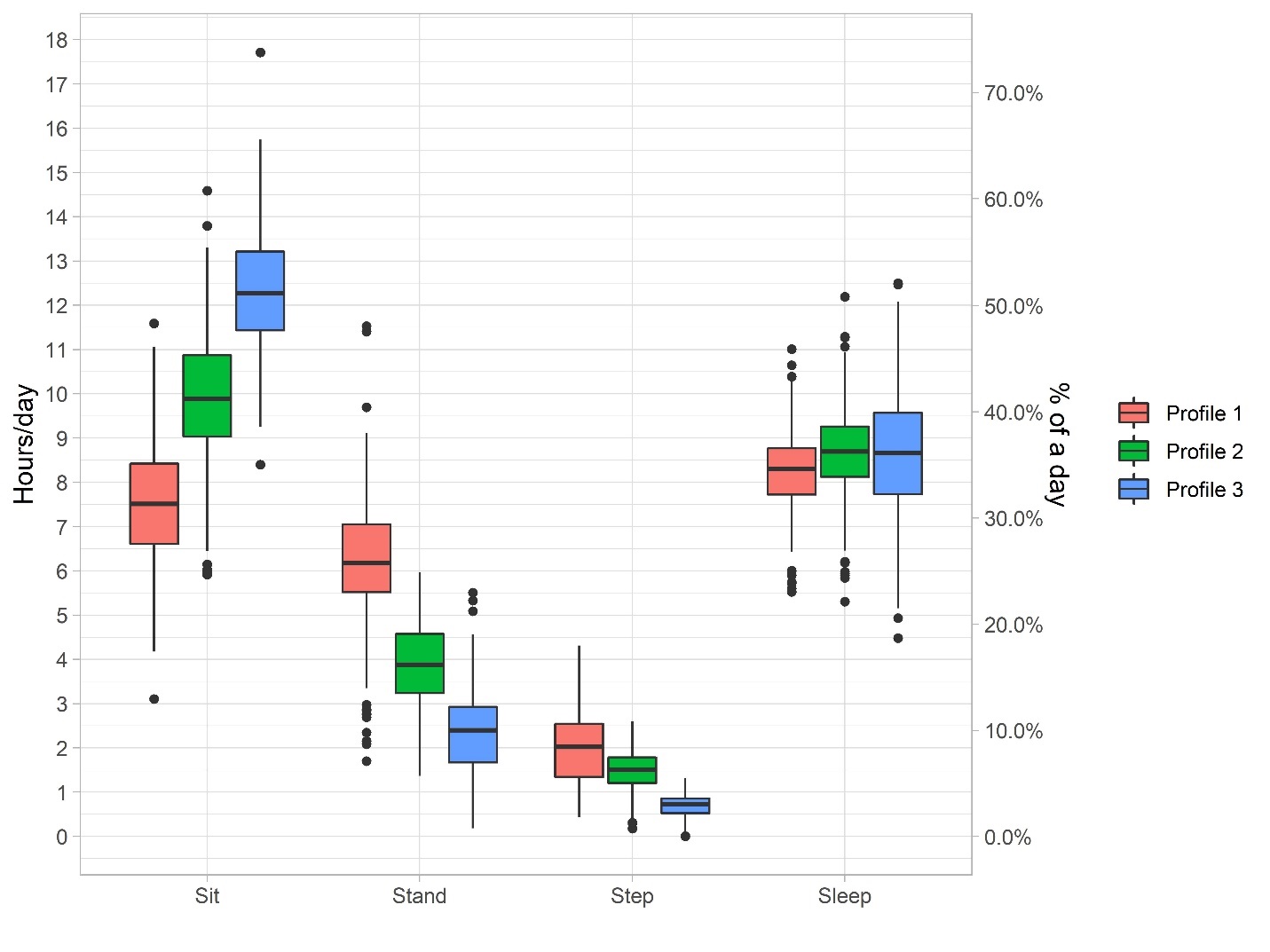
**
